# Supplementary material for: Shared mycorrhizae but distinct communities of other root-associated microbes on co-occurring native and invasive maples
Source: PeerJ. 2019 Jul 30;7:e7295. doi: 10.7717/peerj.7295 (PMC6677121; doi:10.7717/peerj.7295)
Supplement: Supplemental Information 1 [file peerj-07-7295-s001.docx]

Supplematary Table. Summary of two-way ANOVA results for soil chemical parameters

to sampling date, species and their interaction.

|  | Source of Variation | | | | | |
| --- | --- | --- | --- | --- | --- | --- |
|  | Species | | Date | | Species x Date | |
| Variable | F | P | F | P | F | P |
| pH | 0.000 | 0.994 | 0.451 | 0.504 | 0.498 | 0.483 |
| % Organic Matter | 0.204 | 0.653 | 0.046 | 0.830 | 0.181 | 0.672 |
| C/N ratio | 0.855 | 0.358 | 3.192 | 0.078 | 4.281 | **0.042** |
| P mg/kg | 0.175 | 0.677 | 3.095 | 0.083 | 1.492 | 0.226 |
| Ca mg/kg | 0.232 | 0.631 | 0.056 | 0.814 | 0.448 | 0.505 |
| Mg mg/kg | 0.033 | 0.857 | 0.195 | 0.660 | 1.246 | 0.268 |
| K mg/kg | 0.882 | 0.351 | 0.541 | 0.464 | 0.192 | 0.622 |
